# The relationship between refractive error and the risk of diabetic retinopathy: a systematic review and meta-analysis

**DOI:** 10.3389/fmed.2024.1354856

**Published:** 2024-06-04

**Authors:** Yanqing Li, Pengcheng Hu, Li Li, Xianhui Wu, Xi Wang, Yanli Peng

**Affiliations:** ^1^Department of Refractive Surgery, China Aier Eye Hospital Group, Chongqing Aier Eye Hospital, Chongqing, China; ^2^Department of Ophthalmology, The Second Affiliated Hospital of Chongqing Medical University, Chongqing, China; ^3^Department of Refractive Surgery, Dali Aier Eye Hospital, Dali, Yunnan, China

**Keywords:** refractive error, hyperopia, myopia, axial length, diabetic retinopathy, meta-analysis

## Abstract

**Purpose:**

This meta-analysis was conducted to collect all available data and estimate the relationship between refractive error and the risk of diabetic retinopathy (DR) in patients with diabetes, and to assess whether vision-threatening DR (VTDR) is associated with refractive error.

**Methods:**

We systematically searched several literature databases including PubMed, Embase, Cochrane Library, Web of Science, CNKI, CBM, Wan Fang Data, and VIP databases. Pooled odds ratios (OR) and 95% confidence intervals (CI) were calculated using fixed or random effects models. Four models were developed to assess the relationship between refractive error and the risk and DR, VTDR: hyperopia and DR, VTDR; myopia and DR, VTDR; spherical equivalent (SE per D increase) and DR, VTDR; and axial length (AL per mm increase) and DR, VTDR. The included literature was meta-analyzed using Stata 12.0 software, and sensitivity analysis was performed. Publication bias in the literature was evaluated using a funnel plot, Begg's test, and Egger's test.

**Results:**

A systematic search identified 3,198 articles, of which 21 (4 cohorts, 17 cross-sectional studies) were included in the meta-analysis. Meta-analysis showed that hyperopia was associated with an increased risk of VTDR (*OR:* 1.23; 95% *CI*: 1.08–1.39; *P* = 0.001), but not with DR (*OR:* 1.05; 95% *CI*: 0.94–1.17; *P* = 0.374). Myopia was associated with a reduced risk of DR (*OR:* 0.74; 95% *CI*: 0.61–0.90; *P* = 0.003), but not with VTDR (*OR:* 1.08; 95% *CI*: 0.85–1.38; *P* = 0.519). Every 1 diopter increase in spherical equivalent, there was a 1.08 increase in the odds ratio of DR (*OR:* 1.08; 95% *CI*: 1.05–1.10; *P*<0.001), but not with VTDR (*OR:* 1.05; 95% *CI*: 1.00–1.10; *P* = 0.06). AL per mm increase was significantly associated with a decreased risk of developing DR (*OR:* 0.77; 95% *CI*: 0.71–0.84; *P*<0.001**)** and VTDR (*OR:* 0.63; 95% *CI*: 0.56–0.72; *P*<0.001). Analysis of sensitivity confirmed the reliability of the study's findings.

**Conclusion:**

This meta-analysis demonstrates hyperopia was associated with an increased risk of VTDR in diabetes patients. Myopia was associated with a reduced risk of DR. AL is an important influencing factor of refractive error. Every 1 mm increase in AL reduces the risk of DR by 23% and the risk of VTDR by 37%.

**Systematic review registration:**

identifier: CRD42023413420

## 1 Introduction

Based on the data provided by the International Diabetes Federation, the global population of individuals aged 20–79 diagnosed with diabetes in 2017 was estimated to be 424.9 million (1). By the year 2021, the aforementioned figure has experienced an increase, reaching a total of 536.6 million. This implies that the prevalence of diabetes among the global adult population exceeds 10.5% (2). Diabetic retinopathy (DR) is a common microvascular condition associated with diabetes mellitus (3). It ranks as the fifth most prevalent cause of blindness among individuals aged 50 years and above on a global scale (4). Among them, any stage of DR with severe NPDR, PDR, or/and concomitant diabetic macular edema, DME) is defined as vision-threatening diabetic retinopathy (VTDR), which is the main cause of vision impairment in diabetic patients, and it can seriously affect the patient's quality of life. In 2020, the global prevalence of DR was estimated to be 22.27% (103.12 million) and that of VTDR to be 6.17% (28.54 million). By 2045, it is anticipated that the number of patients will rise to 160,5 million and 44,82 million, respectively (5). Monitoring the occurrence of DR, controlling its progression, and reducing the risk of blindness in diabetic patients is an important public health issue.

It is well known that the occurrence and progression of DR are correlated with a longer period of diabetes, higher blood glucose, blood pressure, and serum glycosylated hemoglobin levels (6). In addition to these systemic risk factors, some studies (7, 8) have found that ocular biological parameters can also influence the occurrence and progression of DR, although this assertion is controversial. Lim et al. (7) discovered that diabetic patients with myopic refractive errors and long eye axes had a decreased risk of developing DR, particularly proliferative diabetic retinopathy. However, another prospective cohort study (8) involving 1,370 patients with type 2 diabetes mellitus revealed no correlation between refractive error and the development of DR and VTDR. Longer axial length (AL) and larger axial length/corneal radius (AL/CR) were associated with a significantly lower incidence of DR, but not VTDR, in this study.

Several prior meta-analyses have investigated the relationship between myopia and the risk of DR (9–11). These meta-analyses focused primarily on the association between myopia, AL, and DR risk. According to studies, myopia, and long axial length can decrease the risk of DR. The correlation between hyperopic refractive error and the risk of DR and VTDR, as well as the effect of myopic refractive error and AL on the risk of VTDR, are, however, still equivocal.

New observational studies on the relationship between refractive error and the risk of DR and VTDR have been published in recent years. Therefore, this study conducted a systematic review and meta-analysis to comprehensively assess the degree of association between the two. Understanding this association better will aid in the development of primary prevention strategies for the occurrence of diabetic retinopathy and its severity control, which is crucial for reducing the risk of impaired vision and blindness in diabetic patients.

## 2 Materials and methods

### 2.1 Protocol and guidance

According to the Preferred Reporting Items for Systematic Reviews and Meta-Analyses (PRISMA) statement (12), this systematic review and meta-analysis is registered in PROSPERO, number: CRD42023413420.

### 2.2 Data sources and searches

A systematic search was performed in the following databases: PubMed, Embase, Cochrane Library, Web of Science, CNKI, CBM, Wan Fang Data, and VIP databases. The search was restricted to human studies from database inception to June 2023, with no language restrictions. The reference lists of relevant papers and review articles were also manually searched to find other relevant studies not covered by the original database search. The search terms consist of a combination of subject terms and free words and are tailored to the needs of distinct database searches. Subject headings include “Myopia, Hyperopia, Refractive Error, Axial Length, and Diabetic Retinopathy”; Text words include “Myopia, Nearsightedness”, “Hyperopia, Farsightedness”, “Strabismus, Error, Refraction, Refractive Disorder, Disorder, Refraction”, “Axial Lengths, Eye”, “Eye Axial Length”, “Length, Eye Axial”, “Diabetic Retinopathies”, “Proliferative DR”, “Retinopathies, Diabetic” (Supplementary Table 1).

To minimize bias, the retrieved literature was screened by two trained researchers according to the inclusion and exclusion criteria, first by title and abstract, and then by carefully reading each item of literature that met the requirements of the initial screening. Determination was made regarding the final inclusion of studies, and literature quality assessment and data extraction were performed. Final cross-checks are conducted, and in the event of a dispute, a third-party decision is sought.

### 2.3 Inclusion criteria

Studies were considered acceptable for inclusion if they met the following criteria: (1) Reported are observational studies on the association between refractive error or axial length and the risk of DR or VTDR; (2) The use of DR or VTDR as outcome measures and utilization of standardized protocols[the Early Treatment Diabetic Retinopathy Study (ETDRS) or the Airlie House classification system] to evaluate the severity of DR, VTDR; (3) Spherical equivalent (SE) > 0.5D or > 1.0D was used as the cutoff for hyperopia, while SE −0.5D or −1.0D was used as the cutoff for myopia; (4) reporting odds ratio (OR), risk ratio (RR) and 95% confidence intervals (95% CI) or allowed for the calculation of such metrics from the raw data presented in the article; (5) the moderate quality or higher studies were included.

### 2.4 Exclusion criteria

The exclusion criteria are as follows: (1) Exclude articles with less information if two or more studies share the same case or control subject; (2) No clear definition of myopia thresholds or lack of studies of fundus photography; (3) Studies with incorrect data or data that could not be extracted was excluded; (4) Editorials, letters to the editor, review articles, case reports, conference abstracts, and animal studies were excluded.

### 2.5 Data extraction and quality assessment

Two independent researchers extracted the following information from all eligible studies: first author, year of publication, country, race, age, type of study design, sample size, measurement method of SE, threshold for refractive error, measurement method of AL, type of diabetes, duration of diabetes, diagnostic criteria for DR, and data that either provided OR directly or allowed for its calculation.

Using the Newcastle-Ottawa Scale (NOS), the quality of the included cohort and case-control studies was evaluated. 7 to 9 on the NOS scale indicates high quality, 4 to 6 indicates moderate quality, and 0 to 3 indicates low quality. Using the evaluation criteria recommended by the US Agency for Healthcare Quality and Research (AHRQ), the quality of the included cross-sectional studies was evaluated. The AHRQ evaluation criteria include 11 entries out of a possible 11 points, with 8 to 11 representing high quality, 4 to 7 representing medium quality, and 0 to 3 representing low quality. Two investigators independently evaluated the methodological quality, and a third-party opinion was sought in the event of disagreement.

### 2.6 Definition of DR, VTDR

The severity of diabetic retinopathy was categorized using the modified ETDRS grading scale or the Airlie House classification system as follows: mild nonproliferative diabetic retinopathy (NPDR), moderate NPDR, severe NPDR, and proliferative diabetic retinopathy (PDR). The presence of any degree of DR was categorized as the occurrence of DR. The presence of any stage of DR in conjunction with severe NPDR, PDR, or diabetic macular edema (DME) was defined as the occurrence of VTDR.

### 2.7 Statistical analysis

Data analysis was performed using Stata12.0 software. The odds ratio (*OR*) and 95 % confidence interval (*CI*) were used to evaluate the association between refractive error, axial length, and the risk of DR and VTDR. Combined ORs and 95% CIs were calculated for the following four models: hyperopia and DR, VTDR; myopia and DR, VTDR; spherical equivalent (SE per D increase) and DR, VTDR; AL (per mm increase) and DR, VTDR. If ORs and 95% CIs for different degrees of myopia were provided separately in the literature, or if ORs and 95% CIs for severe NPDR and PDR only were provided in the literature, as well as if ORs and 95% CIs for different types of diabetes were provided in the literature, the ORs for the outcome metrics were combined before their incorporation into Meta-analyses of the corresponding models.

The heterogeneity of results between studies was determined by the *I*^2^ test. Low heterogeneity(*I*^2^ <50%) was modeled using a fixed-effects model, and significant heterogeneity(*I*^2^>50%) was modeled using a random-effects model. The reliability of the overall results was determined by eliminating a single study each time for sensitivity analysis. For models with high heterogeneity, subgroup analysis was used to determine the source of heterogeneity if the number of included studies was less than ten; If the number of included studies exceeded ten, meta-regression and subgroup analysis were employed to determine the source of heterogeneity.

If the number of included articles was less than 10, Egger's test and Begg's test were used to determine whether there was publication bias; If the number of articles included more than 10, a funnel plot combined with Egger's test and Begg's test were used to determine whether there was publication bias. *P*>0.05 suggests no significant publication bias, and *P* < 0.05 suggests possible publication bias. If publication bias exists, proceed to assess the stability of the combined results using the trim and fill method.

## 3 Results

### 3.1 Literature search

**Figure 1:**
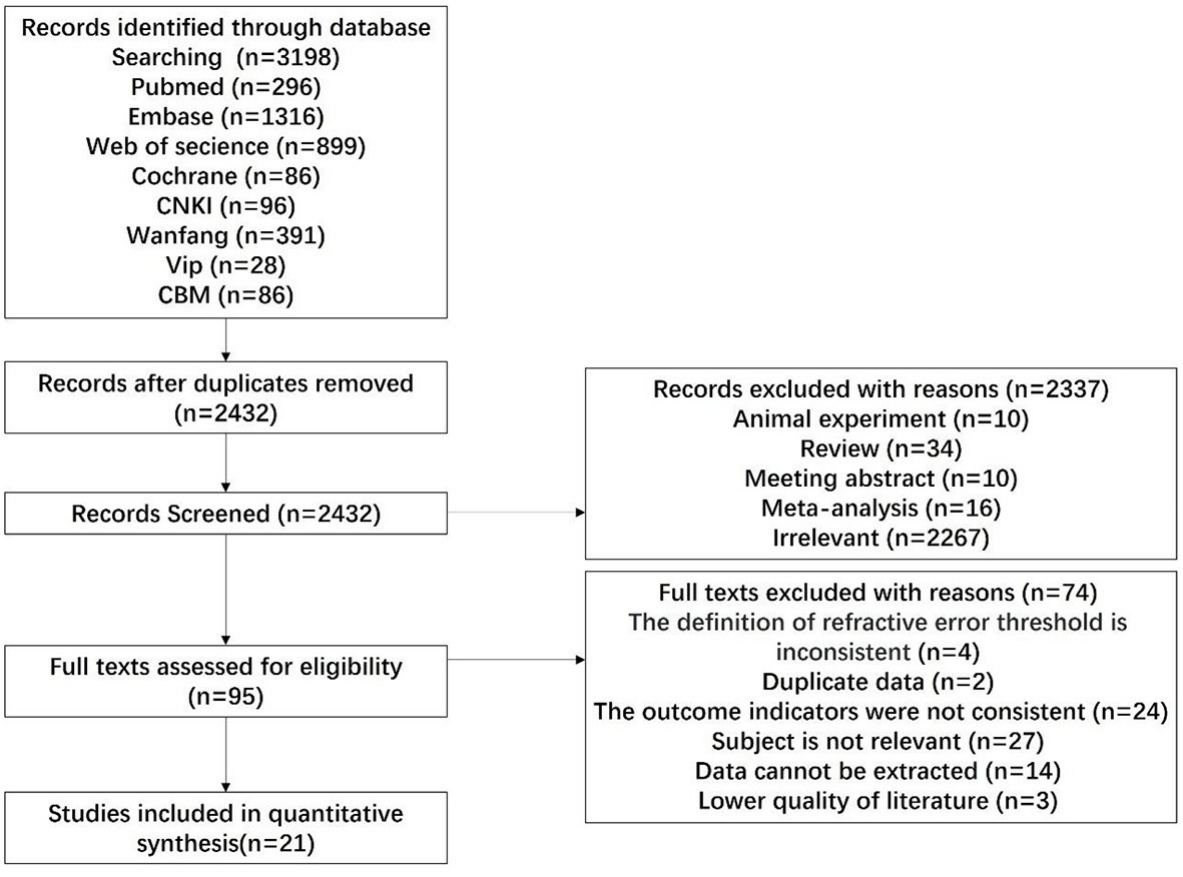
Flow chart of study selection.

Following the search strategy, 3,198 pertinent works of literature were initially identified. Following the exclusion of duplicate literature (*n* = 766), articles failing to satisfy the inclusion criteria (*n* = 2,432) were systematically excluded through a meticulous examination of the titles and abstracts present in the remaining literature. Twenty-one articles were finally included in the literature. The specific process and results of the literature screening are shown in Figure 1.

### 3.2 Study characteristics

Among the 21 articles, there were four cohort studies, and the average score of NOS was 7.5 points (Table 1). There were 17 cross-sectional studies (11 population-based cross-sectional studies and 6 clinical cross-sectional studies). The average score of AHRQ was 6.88 (Table 2).

**Table 1:** Basic characteristics and quality evaluation of cohort studies.

**References**	**Country**	**Ethnicity**	**Age range/ mean age**	**Study design**	**Participants**	**SE measurement method**	**Refractive status**	**AL measurement method**	**Diabetes (types)**	**Diabetes (duration)**	**DR grading method**	**NOS**
Xu et al. (13)	China	Asian	≥40	Cohort study	2,602	AR-610	/	Lensstar 900	/	/	ETDRS	8
Man et al. (14)	Singapore	Asian	40–80	Cohort study	840	RK-5	SE>0.5D SE <-0.5D	IOL Master	/	/	Airlie House	8
Thakur et al. (15)	India	Asian	6–90	Cohort study	9,813	/	SE>0.5D SE <-0.5D	/	Type 1 Type 2	Type 1 (11–18) Type 2 (5–7)	ETDRS	6
Lin et al. (16)	China	Asian	≥30	Cohort study	1,817	AR-610	SE <-5.0D	/	Type 1 Type 2	/	Airlie House	8

**Table 2:** Basic characteristics and quality evaluation of cross-sectional studies.

**References**	**Country**	**Ethnicity**	**Age range/ mean age**	**Study design**	**Participants**	**SE measurement method**	**Refractive status**	**AL measurement method**	**Diabetes (types)**	**Diabetes (duration)**	**DR grading method**	**AHRQ**
Xie et al. (17)	China	Asian	45–89	Cross-sectional, Population based	362	Neitz CT-R Camera	/	/	/	/	ETDRS	7
Lim et al. (7)	Singapore	Asian	40–80	Cross-sectional, Population based	629	RK-5	SE>0.5D SE <-0.5D	IOLMaster	/	/	Airlie house	7
Man et al. (18)	Australia	Caucasian	≥18	Cross-sectional, Population based	367	Retinomax 2	SE>0.5D SE <-0.5D	IOLMaster	Type 1 Type 2	10	ETDRS	8
Ganesan et al. (19)	India	Asian	≥40	Cross-sectional, Population based	1,058	Beta 200	SE>0.5D SE <-0.5D	A-scan ultrasonography	Type 2	/	ETDRS	7
Jee et al. (20)	Korea	Asian	≥40	Cross-sectional, Population based	1,678	/	SE>1.0D SE <-1.0D	/	Type 1 Type 2	/	ETDRS	6
Pan et al. (21)	Singapore	Indian	40–84	Cross-sectional, Population based	3,400	RK-5	SE>0.5D SE <-0.5D	IOLMaster	/	/	Airlie House	8
Chao et al. (22)	Korea	Asian	≥40	Cross-sectional, Population based	1,685	KR-8800	SE>1.0D SE <-1.0D	/	/	/	ETDRS	7
He et al. (23)	China	Asian	34–97	Cross-sectional, Population based	2,057	KR-8800	SE>0.5D SE <-0.5D	IOLMaster	Type 2	/	ETDRS	7
Wang et al. (24)	China	Asian	26–83	Cross-sectional, Population based	1,096	/	/	Lenstar 900	Type 2	/	ETDRS	7
Bikbov et al. (25)	Russia	Russian	40–94	Cross-sectional, Population based	5,105	HRK-7000A	/	/	Type 1 Type 2	4.74 ± 8.01	ETDRS	7
Wang et al. (26)	China	Asian	30–85	Cross-sectional, Population based	1,838	KR-8800	SE>0.5D SE <-0.5D	Lenstar LS900	Type 2	9.0 ± 7.0	ETDRS	7
Yang et al. (27)	China (Taiwan)	Asian	19–85	Cross-sectional, clinic based	166	/	/	A-scan ultrasonography	/	≥10	ETDRS	7
Jiang et al. (28)	China	Asian	29–80	Cross-sectional, clinic based	118	/	SE>0.5D SE <-0.5D	IOLMaster	/	≥10	ETDRS	6
Man et al. (29)	Australia	Caucasian	56	Cross-sectional, clinic based	85	/	/	IOLMaster	Type 1 Type 2	/	ETDRS	8
Yang et al. (30)	China	Asian	≥18	Cross-sectional, clinic based	327	KR-8801	SE>0.5D SE <-0.5D	A-scan ultrasonography	Type 2	≥6	ETDRS	6
Qin et al. (31)	China	Asian	35–70	Cross-sectional, clinic based	102	/	/	IOLmaster	Type 2	10-15	ETDRS	6
Shehab et al. (32)	Iraq	Asian	≥43	Cross-sectional, clinic based	221	TomeyUSA /RC500	SE>1.0D SE <-1.0D	A-scan ultrasonography	Type 2	≥5.5	ETDRS	6

### 3.3 Association between hyperopia and risk of DR, VTDR

**Figure 2:**
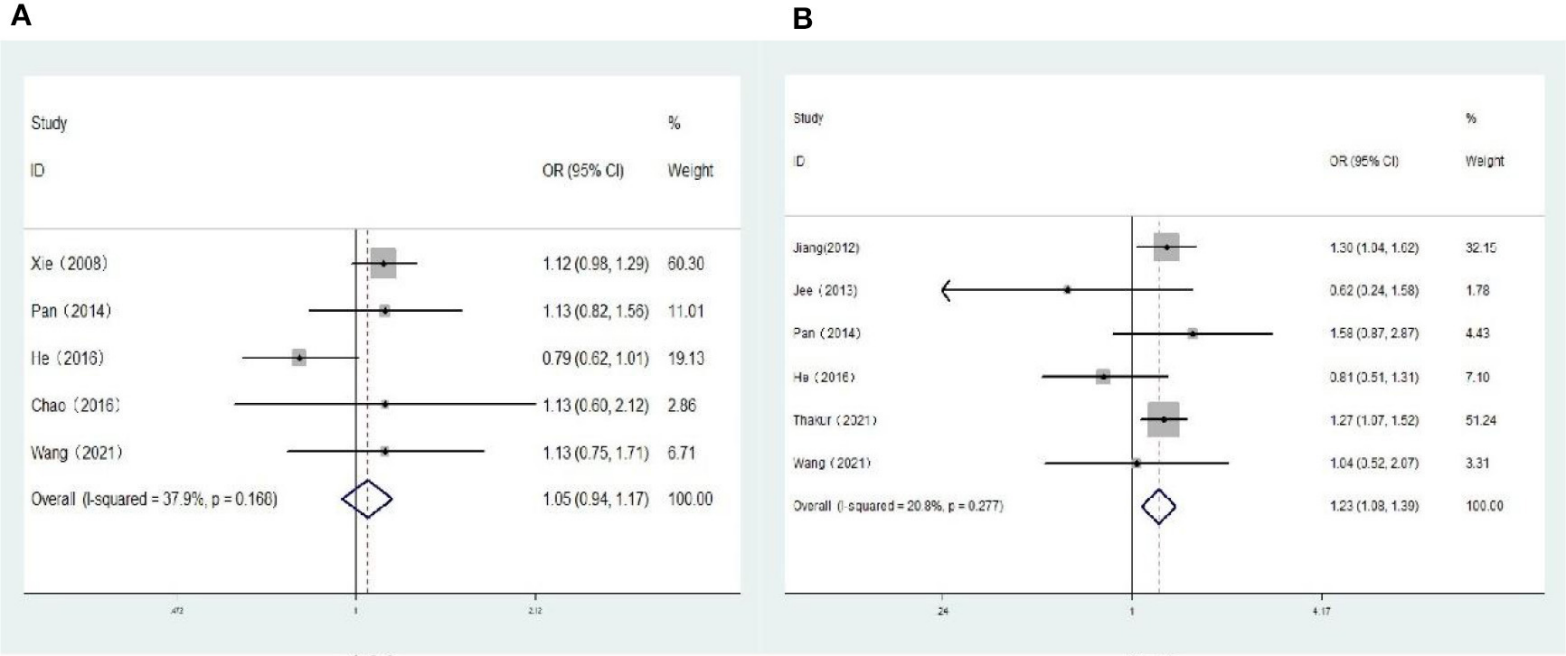
**(A)** Forest plot of the association between hyperopia and DR risk. **(B)** Forest plot of the association between hyperopia and VTDR risk.

Five population-based cross-sectional studies reported the association between hyperopia and the risk of DR. One cohort study and five population-based cross-sectional studies reported the association between hyperopia and the risk of VTDR. The heterogeneity test results showed that the heterogeneity was low, so the fixed-effects model was used for Meta-analysis. The findings revealed that there was no statistically significant correlation between hyperopia and the risk of DR (*OR:* 1.05; 95% *CI*: 0.94–1.17; *P* = 0.374; *I*^2^=37.9%; Table 3, Figure 2A). Nevertheless, hyperopia was significantly correlated positively with the risk of VTDR(*OR:* 1.23; 95% *CI*: 1.08–1.39; *P* = 0.001; *I*^2^= 20.8%; Table 3, Figure 2B). It is suggested that hyperopia may be a risk factor affecting the severity of diabetic retinopathy.

**Table 3:** Meta-analysis of the correlation between refractive error and DR and VTDR risk.

**Index**	**Model types**	**Quantity**	**Heterogeneity test**		**Effects models**	**Meta-analysis results**		**Publication bias**		
			***I** ^2^*	*P*		***OR*** **(95%*****CI*****)**	*P*	**Funnel plot**	**Begg's test**	**Egger's test**
Hyperopia	Hyperopia and DR	5	37.9	0.168	Fixed- effect	1.05 (0.94,1.17)	0.374	/	0.462	0.845
	Hyperopia and VTDR	6	20.8	0.277	Fixed- effect	1.23 (1.08,1.39)	0.001	/	0.707	0.227
Myopia	Myopia and DR	8	50.7	0.048	Random- effect	0.74 (0.61,0.90)	0.003	/	0.174	0.198
	Myopia and VTDR	5	0	0.766	Fixed- effect	1.08 (0.85,1.38)	0.519	/	0.806	0.900
SE (per D increase)	SE and DR	7	25.4	0.235	Fixed- effect	1.08 (1.05,1.10)	<0.001	/	0.072	0.064
	SE and VTDR	5	0	0.667	Fixed- effect	1.05 (1.00,1.10)	0.06	/	1.000	0.437
AL (per mm increase)	AL and DR	14	59.3	0.002	Random- effect	0.77 (0.71,0.84)	<0.001	Asymmetry	0.016	0.002
	AL and VTDR	8	0	0.476	Fixed- effect	0.63 (0.56,0.72)	<0.001	/	0.902	0.993

P <0.1 considered high heterogeneity.

P <0.05 represents a statistical difference.

### 3.4 Association between myopia and risk of DR, VTDR

**Figure 3:**
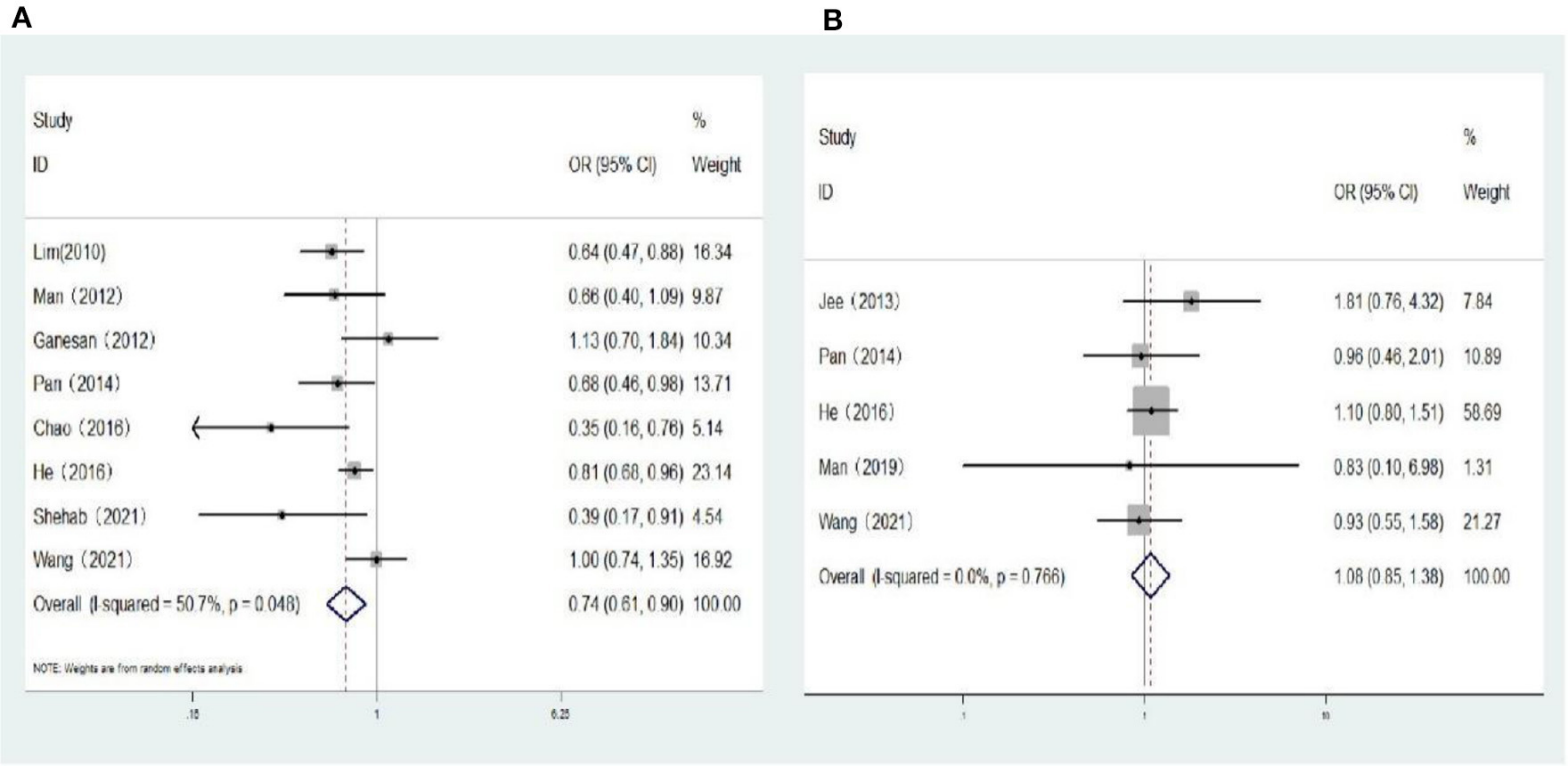
**(A)** Forest plot of the association between myopia and DR risk. **(B)** Forest plot of the association between myopia and VTDR risk.

Seven population-based cross-sectional studies and one clinic-based cross-sectional study reported the association between myopia and the risk of DR. The results of the heterogeneity test indicated the presence of significant heterogeneity, so we chose the random-effects model. The results showed that there was a negative correlation between myopia and the risk of DR (*OR:* 0.74; 95% *CI*: 0.61-0.90; *P* = 0.003; *I*^2^=50.7%; Table 3, Figure 3A). Further exploration of the sources of heterogeneity is needed.

One cohort study and four population-based cross-sectional studies reported the association between myopia and the risk of VTDR. The heterogeneity test results showed that the heterogeneity was low, so the fixed-effects model was used for Meta-analysis. The results showed that there was no significant correlation between myopia and the risk of VTDR (*OR:* 1.08; 95% *CI*: 0.85–1.38; *P* = 0.519; *I*^2^= 0%; Table 3, Figure 3B).

### 3.5 Association between spherical equivalent (per D increase) and risk of DR, VTDR

**Figure 4:**
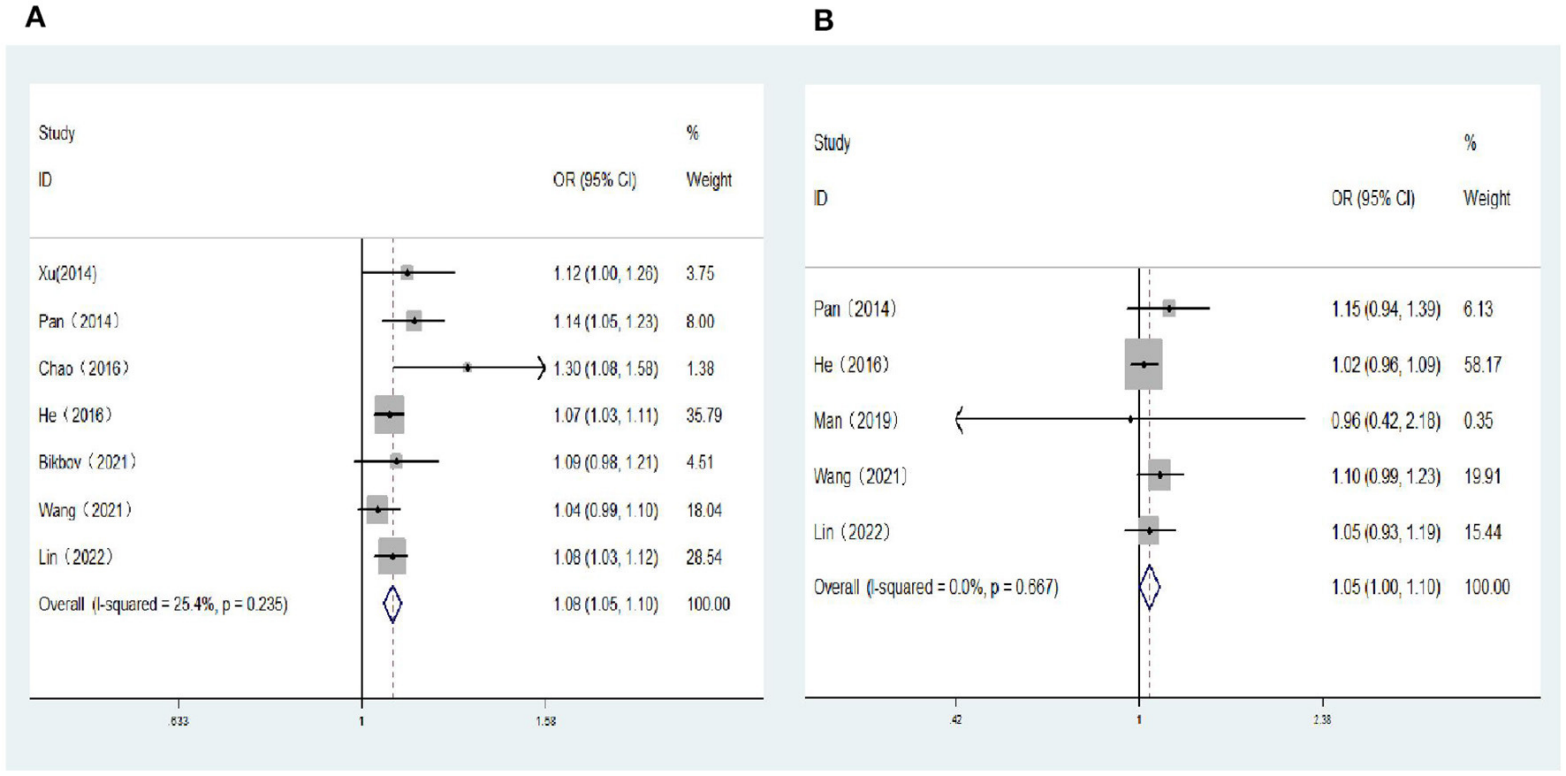
**(A)** Forest plot of the association between spherical equivalent (per D increase) and DR risk. **(B)** Forest plot of the association between spherical equivalent (per D increase) and VTDR risk.

Five population-based cross-sectional studies and two cohort studies reported the association between SE (per D increase) and the risk of DR. Two cohort studies and three population-based cross-sectional studies reported the association between SE (per D increase) and the risk of VTDR. The heterogeneity test results showed that the heterogeneity was low, so the fixed-effects model was used for Meta-analysis. The results showed a positive correlation between SE (per D increase) and the risk of DR (*OR:* 1.08; 95% *CI*: 1.05–1.10; *P* < 0.001; *I*^2^= 25.4%; Table 3, Figure 4A), and no significant correlation with the risk of VTDR (*OR:* 1.05; 95% *CI*: 1.00–1.10; *P* = 0.06; *I*^2^=0%; Table 3, Figure 4B).

### 3.6 Association between axial length (per mm increase) and risk of DR, VTDR

**Figure 5:**
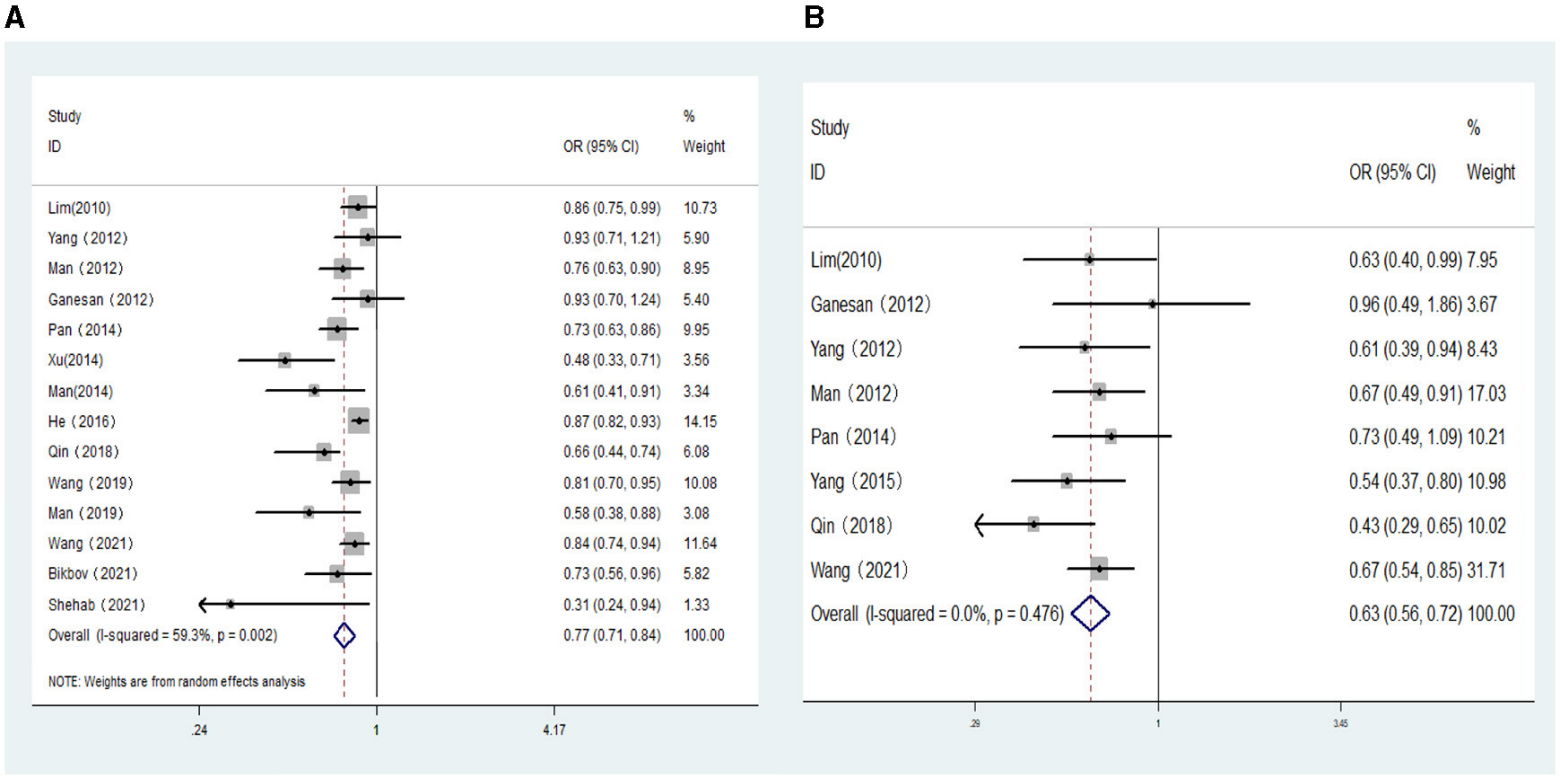
**(A)** Forest plot of the association between axial length (per mm increase) and DR risk. **(B)** Forest plot of the association between axial length (per mm increase) and VTDR risk.

Eight population-based cross-sectional studies, four clinical-based cross-sectional studies, and two cohort studies reported the association between AL (per mm increase) and the risk of DR. The results of the heterogeneity test indicated the presence of significant heterogeneity, so we chose the random-effects model. The results showed that AL (per mm increase) was negatively correlated with the risk of DR (*OR:* 0.77; 95% *CI*: 0.71–0.84; *P* < 0.001; *I*^2^= 59.3%; Table 3, Figure 5A). Further exploration of the sources of heterogeneity is needed.

Six population-based cross-sectional studies and four clinical-based cross-sectional studies reported the association between AL (per mm increase) and the risk of VTDR. The results of the heterogeneity test indicated the presence of significant heterogeneity, so we chose the random-effects model. The results showed that AL (per mm increase) was negatively correlated with the risk of VTDR(*OR:*0.59; 95% *CI:*0.42–0.82; *P* < 0.001; *I*^2^= 91.9%). By conducting sensitivity analysis, it was found that the primary sources of heterogeneity were two articles by He (23) and Shehab (32). After excluding these two studies, the heterogeneity of the combined OR value decreased significantly, and the analysis results still suggested that AL (per mm increase) was negatively correlated with the risk of VTDR (*OR:* 0.63; 95% *CI*: 0.56–0.72; *P* < 0.001; *I*^2^= 0%; Table 3, Figure 5B).

### 3.7 Sensitivity analysis

Sensitivity analyses were undertaken by removing one study at a time in each study model to verify the stability of the combined results. The results of sensitivity analysis showed that the removal of a single study in each model had little effect on the total combined effect, and the pooled OR value was stable (Supplementary Figure 1).

### 3.8 Meta-regression

In the model of AL (per mm increase) and the risk of DR, many literatures were included, and the heterogeneity test results suggested that there was high heterogeneity. Therefore, One-way Meta-regression analysis was used to analyze the causes of heterogeneity. Although the ocular axis measurement instrument, the type of diabetes, and the duration of diabetes may be sources of heterogeneity, the heterogeneity caused by these factors cannot be analyzed because some literature does not explicitly mention relevant information. Finally, three factors including publication year, race, and research type were included for screening. The results showed that the heterogeneity may be related to different types of studies (*P* < 0.05), as shown in Table 4.

**Table 4:** Results of one-way meta-regression analysis.

**Factor**	**Regression coefficient**	**Standard error**	***t***	***P***
Publication year	−0.0181	0.0734	−0.25	0.810
Race	−0.1073	0.0933	−1.15	0.276
Research type	−0.2053	0.0717	−2.86	0.017

### 3.9 Subgroup analyses

Subgroup analysis within the myopia and DR risk model was conducted based on the myopia threshold (Table 5, Supplementary Figure 2). In the SE <−0.5D subgroup, myopic ametropia was associated with a lower risk of DR (*OR:* 0.80; 95% *CI*: 0.68-0.94; *P* = 0.006; *I*^2^= 31.7%); In the SE <−1.0D subgroup, myopic refractive error was also associated with a lower risk of DR(*OR:* 0.37; 95% *CI*: 0.21–0.65; *P* = 0.001; *I*^2^= 0%). The heterogeneity of the two subgroups was not significant, suggesting that the threshold of myopia definition may be a significant influencing factor of heterogeneity.

**Table 5:** Results of myopia and DR subgroup analysis.

**Basis**	**Outcome index**	**Quantity**	**Heterogeneity test**		**Effects models**	**Meta-analysis results**	
			***I** ^2^* **(%)**	*P*		***OR*** **(95%CI)**	*P*
Myopia threshold	SE <-0.5D	6	31.7	0.198	Random- effect	0.80 (0.68,0.94)	0.006
	SE <-1.0D	2	0	0.853	Random- effect	0.37 (0.21,0.65)	0.001

P <0.1 considered high heterogeneity.

P <0.05 represents a statistical difference.

In the model of AL (per mm increase) and DR risk, three subgroups were obtained based on the type of study as the basis for grouping (Table 6, Supplementary Figure 3). Longer AL was associated with a lower risk of DR in the population-based cross-sectional study subgroup (*OR:* 0.84; 95% *CI*: 0.80–0.88; *P* < 0.01; *I*^2^= 5.1%) and cohort study subgroup (*OR:* 0.52; 95% *CI*: 0.39-0.69; *P* < 0.01; *I*^2^= 0%). The two remained negatively correlated in the subgroup of clinic-based cross-sectional studies, but with significant heterogeneity (*OR:* 0.64; 95% *CI*: 0.46–0.90; *P*=0.01; *I*^2^= 71.3%). This suggests that clinical-based cross-sectional studies may be the source of heterogeneity in the model.

**Table 6:** Results of AL and DR subgroup analysis.

**Basis**	**Outcome index**	**Quantity**	**Heterogeneity test**		**Effects models**	**Meta-analysis results**	
			***I**^2^* **(%)**	*P*		***OR*** **(95%CI)**	*P*
Myopia threshold	Cross-sectional, population based	8	5.1	0.391	Random-effect	0.84 (0.80,0.88)	<0.001
	Cross-sectional, clinic based	4	71.3	0.015	Random-effect	0.64 (0.46,0.90)	0.01
	Cohort study	2	0	0.514	Random-effect	0.52 (0.39,0.69)	<0.001

P <0.1 considered high heterogeneity.

P <0.05 represents a statistical difference.

### 3.10 Publication bias

**Figure 6:**
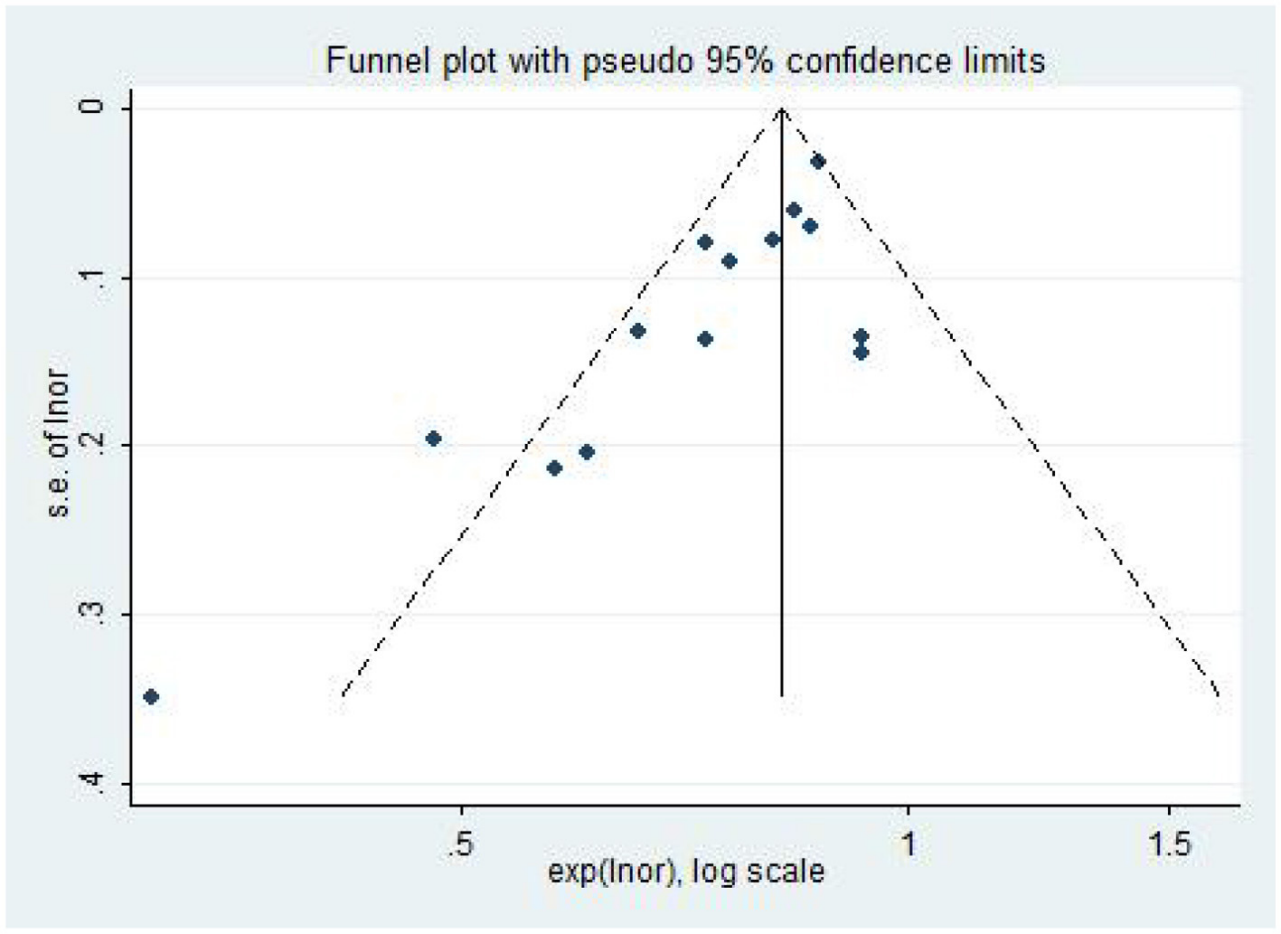
The funnel plot of the association between AL (per mm increase) and DR risk.

The included literature was subjected to publication bias testing using Stata 12.0 software. In the model of AL (per mm increase) and the risk of DR, a funnel plot combined with Egger's test and Begg's test were used to determine whether there was publication bias, and the rest of the models were tested using only the Egger's test and Begg's test. The analysis results (Table 3) showed that there may be publication bias in the model of AL and DR risk (The funnel plot is asymmetric, Figure 6; Bgge's test *P* = 0.016; Egger's test *P* = 0.002), and no significant publication bias is found in the other models. Then, the “trim and fill” method was used and no correction was made to the original estimates, which further proved that the results were stable (Supplementary Figure 4).

## 4 Discussion

In this systematic review and meta-analysis, the relationship between refractive error and the risk of DR and VTDR is explored in several dimensions: hyperopia and DR, VTDR; myopia and DR, VTDR; SE (per D increase) and DR, VTDR; AL (per mm increase) and DR, VTDR. During the literature screening process, we found that both Jee et al. (20) and Chao et al. (22) analyzed the correlation between refractive error and the risk of DR based on the 2008–2011 Korea National Health and Nutrition Survey (KNHNS) database. Chao's data was deemed more comprehensive and thus Jee's data was omitted from the analysis. Similarly, the study subjects recruited for the articles by Wang et al. (8) and Wang et al. (26) were all type 2 diabetic patients of the same age group in Guangzhou, China. As a result, following a comparison of data integrity, the study by Wang et al. (8) was excluded from the meta-analysis to prevent an increased risk of bias. In the end, 21 observational investigations were incorporated.

The results of the Meta-analysis showed that diabetic patients with myopia had a lower risk of developing DR and that AL (per mm increase) was significantly associated with a decreased risk of DR, VTDR. This finding aligns with the results reported in multiple prior meta-analyses (9–11). The current meta-analysis validates and expands upon the discovery. In contrast to the aforementioned meta-analysis, this investigation expanded the overall sample size, augmented the number of studies incorporated in the analysis twofold, and conducted subgroup analyses and meta-regression to assess the stability of the observed associations. Furthermore, this study found for the first time by meta-analysis that hyperopia may increase the risk of VTDR in diabetic patients. The correlation between SE (per D decrease) and the risk of DR was analyzed by Wang et al. (10). However, due to the limited number of included studies and the high level of heterogeneity, accurate findings were not available. The utilization of the SE (per D increase) concept was observed to be more prevalent in several studies (21–23). Therefore, we have incorporated more literature into this paper to investigate the association between SE (increase per D) and the risk of DR. According to the study's findings, for every 1 diopter increase in spherical equivalent, there was a 1.08 increase in the odds ratio of DR; Thus, this paper proposes that the impact of refractive error on the risk of DR occurrence is primarily seen in the lower risk of DR associated with myopic refractive error. Similarly, the effect of refractive error on the risk of VTDR occurrence is mainly observed in the higher risk of VTDR associated with hyperopic refractive error. Nevertheless, this study did not discover any association between hyperopia and DR, or between myopia and the likelihood of developing VTDR. This lack of correlation could be attributed to variations in the criteria used to define refractive error in different studies, as well as most studies that measured non-ciliary body paralyzed refractive status in adults. It is also possible that the pathophysiology of DR differs among races and is not affected by refractive state. Further rigorous research is required to investigate the potential association between the two in the future.

There is some heterogeneity in some of the research models in this study. A subgroup analysis in the model of myopia and DR risk revealed that heterogeneity may result from varying thresholds for the definition of myopia across studies. Meta-regression indicates that, in the model of AL (per mm increase) and DR risk, clinic-based cross-sectional studies might be the primary source of heterogeneity. In the model of AL (per mm increase) and VTDR risk, sensitivity analyses found the articles by He et al. (23) and Shehab et al. (32) to be the main source of heterogeneity. Twenty-five hundred diabetic patients were included in the study by He et al. (23), but only 70 eyes developed severe NPDR or PDR. There was no significant correlation between AL (per mm increase) and VTDR risk, according to their studies. Fewer cases of VTDR included may lead to heterogeneity of results. Shehab et al. (32) classified DR severity into the following categories in their study: mild NPDR, moderate NPDR, severe NPDR, and PDR. We combined the ORs of the severe NPDR and PDR among them and included the pooled OR in the meta-analysis. This pooled OR was significantly lower than those of other studies, which may be one of the reasons why this study was a source of heterogeneity. In addition, the instrument used to measure AL in this study was contact ultrasound A-scan. One study (33) found a statistically significant difference in AL measured by contact ultrasound A-scan and IOL Master in eyes with clinically significant macular edema (CSME). Ultrasound A-scan may be affected by pathologic thickening of the retina, resulting in a shorter measured axial length than IOL Master. IOL Master is more accurate in measuring axial length (27). These differences may also have led to heterogeneity. By eliminating these two studies, the heterogeneity of the pooled OR was substantially reduced, while the pooled outcomes remained unchanged. Thus, the present investigation still supports longer AL is protective against VTDR.

Research conducted since the 1960s has indicated that myopia might serve as a protective factor against DR (34). Based on the analysis of the KNHNS database, Chao et al. (22) found that myopia was associated with a lower risk of DR and VTDR. Several studies (14, 35) have found, however, that myopia does not contribute to the onset or progression of DR. The observed variations in results could potentially be ascribed to disparities in the definitions and categorizations of myopia and diabetic retinopathy across various research investigations, insufficient sample sizes, variations in refractive status measurement instruments, and disparities in race. Presently, reports on the correlation between refractive error and DR and its severity concentrate primarily on the effect of myopia on the risk of DR, whereas reports on the correlation between hyperopia and the risk of DR and its severity are scarce. According to the findings of the Beijing Eye Study (17), hyperopia is the primary ocular factor associated with the development and progression of DR in Chinese individuals aged 45 and older. Hainsworth et al. (36) also found that hyperopia is an independent risk factor for PDR. However, no article has been published on Meta-analysis of this. The pooled results of this paper show that the risk of VTDR in diabetic patients with hyperopia is 1.23 times that in diabetic patients with emmetropia. This suggests that hyperopia might serve as a risk factor for the severity of DR. For the prevention of further visual impairment, it is advised that this population undergo routine screening for fundus DR, early detection of VTDR, and treatment thereof.

From age 20–60, the refractive state of the eye changes with age, showing a decrease in the proportion of myopia and an increase in the proportion of hyperopia (37). However, higher HbA1c levels in diabetic patients increase the risk of myopia (38). This may be due to changes in the refractive index of the lens (39) or affected by choroidal blood perfusion (38), diabetic patients will have myopia offset, resulting in fluctuations in the refractive state. This may also be one of the reasons why some of the current clinical articles have found that diabetic patients with hyperopia are at a higher risk of developing VTDR. In contrast, the axial length of adults remains relatively stable and is less affected by blood glucose fluctuations. Previous research has indicated (18) that the protective effect of myopia against DR is largely attributable to the longer AL, whereas other refractive components make negligible contributions to this correlation. Refractive myopia and hyperopia were likely surrogates for AL. Thus, when assessing the ocular risk factors of DR in diabetic patients, AL should be the primary and most direct indicator; it may be the principal pathophysiological driver of the correlation between ametropia and the severity and risk of DR.

The independent association between AL (per 1 mm increase) and a lower risk of DR and VTDR suggests that AL may serve as a protective factor against the development and severity of DR. Consistent with the results of a previous study (26). The Kailuan eye study, which comprised a substantial sample size of 1,096 diabetic patients, observed that the incidence of DR decreased by 19% for each 1 mm increase in AL (24). This is similar to the results of the analysis in this study. This paper demonstrates, through a synthesis of the available evidence evaluating the correlation between ocular axis length (AL) and the risk of DR and VTDR, that the risk of VTDR is reduced by 37% and the risk of DR is diminished by 23% for each 1 mm increase in AL. Several population-based studies (18, 27) also indicated that each millimeter increase in AL was significantly associated with a decreased risk of DR, VTDR. Man et al. (14) found a correlation between AL and the incidence of DR; however, they did not identify any statistically significant correlation between AL and the incidence of VTDR. This may be attributable to the fact that, during follow-up, fewer cases developed VTDR. It is suggested that future studies may increase the number of cases included in the VTDR to improve the accuracy of the findings.

The precise mechanism through which refractive error influences the risk of DR and its severity remains unknown. Given the significant role of AL to refractive error (40), the majority of hypotheses revolve around pathological alterations linked to AL. The first hypothesis: Prior research has established that hyperopia is associated with a thicker retina (41). The axial length of myopia is longer, the choroid and retina will become thinner, and there will be different degrees of atrophy and degeneration (42). At this time, the velocity of retinal arterioles decreases (43), the pressure attenuates (44), and the diameter of retinal vein blood vessels becomes smaller (16), which in turn leads to a decrease in retinal blood flow. The hypoperfused state reduces the metabolic demands on the retina, mitigating the hypoxic response necessary for DR and reducing the risk of DR. The second hypothesis: vascular endothelial growth factor (VEGF) is implicated in the development of DR in juvenile patients with diabetes, as well as in adult patients with type 1 diabetes (T1DM) and type 2 diabetes (T2DM), according to clinical evidence (45). VEGF has been recognized as the most potent factor in promoting both physiological and pathological angiogenesis. This can result in the proliferation and migration of endothelial cells, as well as an increase in vascular permeability (46). Persons with hyperopia and short eye axes have been reported to have elevated levels of intravitreal VEGF (47). Such elevated VEGF concentrations could potentially worsen the symptoms of DR and facilitate the progression of VTDR. An increase in AL, on the other hand, decreased the concentration of VEGF in the vitreous cavity of diabetic patients, thereby decreasing the risk of DR (48, 49). The third hypothesis pertains to posterior vitreous detachment (PVD). It was discovered that patients with PVD had a reduced incidence of PDR compared to those without PVD (50). The protective mechanism may be since VEGF concentration in the vitreous is lower in patients with PVD (51) or that PVD prevents retinal or optic disc neovascularization in patients with DR (50), increasing the diffusion of oxygen in the vitreous (52). By increasing the incidence of PVD (53), myopia or a long ocular axis decreases the risk of DR. There is no significant association between hyperopia and PVD (19). Vitreous-retinal detachment in hyperopia develops at a relatively sluggish rate, and the exacerbation of hypoxia in the inner retina due to delayed vitreous detachment increases the severity of DR (36). The fourth hypothesis posits that transthyretin (TTR), which is abundant in the vitreous of diabetic patients with myopia and is synthesized and secreted by retinal pigment epithelial cells (54), is substantially associated with the risk and severity of diabetic retinopathy (DR). TTR may reduce the risk of DR by affecting the vitreous contents of key factors in the Tie2 pathway for neovascularization (54). The impact of refractive error on the risk and severity of DR may not be the result of a single mechanism operating in isolation but rather is influenced by a multitude of mechanisms. Additional research is required to comprehend the implications of different refractive states on the vascular morphology of the retina and choroidal tissue, as well as to elucidate their relationship with the risk of DR and VTDR.

Our meta-analysis has some limitations that need to be improved. First of all, due to the predominantly cross-sectional design of the studies incorporated, a definitive causal relationship for the observed associations could not be established. Massive prospective studies are required to elucidate the intricate causal connections between the two; Second, across all the studies incorporated in this article, the thresholds for defining myopia and hyperopia were not standardized, and the majority of the studies assessed non-ciliary paralysis refractive status in adults. These factors may add to the instability of the study results; Third, differences in Ocular Biometric Parameters measured by different devices, which may affect results; Finally, Effective subgroup discussions were not possible due to unclear descriptions of the type and duration of diabetes in some of the included studies. This may be another important source of heterogeneity in this study, and it is recommended that future correlation studies should provide clarity on the type and duration of diabetes in study participants.

Despite these limitations, the present Meta-analysis is importantly innovative: First, adopting more rigorous criteria for the inclusion and exclusion of literature to enhance the dependability of meta-analyses; Second, a total of 21 articles were included in this Meta-analysis, with large sample size and a high overall literature quality; Third, we conducted a comprehensive review of the effects of hyperopia, myopia, SE (increase per D), and AL (increase per mm) on the risks of DR and VTDR. The risk correlation model between hyperopia and VTDR, SE (increase per D), and DR, VTDR was established for the first time. The findings derived from the meta-analysis will offer novel insights into the correlation between refractive error and DR, VTDR; Fourth, the origins of heterogeneity were explored through the application of sensitivity analysis, meta-regression, and subgroup analysis; finally, the publication bias of the pooled results of the model of AL (per mm increase) and DR risk was further analyzed using the trim and fill method. There was no reversal of the pooled results before and after clipping and patching, which further proved that the results were stable. As a consequence, the findings of this research are highly reliable.

## 5 Conclusion

In this meta-analysis of the largest and latest observational study, we found that hyperopia is a risk factor for VTDR in diabetic patients. These findings have implications for clinical practice and public health. It is advisable that individuals in this category undergo routine screening for fundus DR, and early detection and treatment of VTDR are implemented to prevent the progression of visual impairment. Myopia is associated with a lower risk of DR. Every 1 diopter increase in spherical equivalent, there was a 1.08 increase in the odds ratio of DR. AL is an important influencing factor of refractive error. Longer axial length was associated with a significantly lower risk of DR and VTDR. For every 1 mm increase in AL, the risk of DR decreased by 23%, and the risk of VTDR decreased by 37%. More high-quality studies are needed in the future to provide accurate, objective, and evidence-based explanations for the complex causal links between refractive error and the risk of DR and VTDR. To prevent or delay the onset and progression of DR in diabetic patients with varying refractive states and eye axis lengths by establishing a screening system for stratified assessment of DR and VTDR.

## Data availability statement

The original contributions presented in the study are included in the article/Supplementary material, further inquiries can be directed to the corresponding author.

## Author contributions

YL: Methodology, Software, Writing—original draft. PH: Data curation, Software, Writing—original draft. LL: Supervision, Writing—review & editing. XWu: Methodology, Supervision, Writing—review & editing. XWa: Software, Validation, Writing—review & editing. YP: Data curation, Methodology, Software, Writing—review & editing.
