# Supplementary material for: The relationship between refractive error and the risk of diabetic retinopathy: a systematic review and meta-analysis
Source: Front Med (Lausanne). 2024 Jun 4;11:1354856. doi: 10.3389/fmed.2024.1354856 (PMC11183799; doi:10.3389/fmed.2024.1354856)
Supplement: Supplementary file 1 [file Data_Sheet_1.docx]

Supplementary Material

This document supplements the manuscript entitled “The Relationship Between Refractive Error and The Risk of Diabetic Retinopathy : A Systematic Review and Meta-Analysis”

# Supplementary Figures and Tables

## Supplementary Figures

**Supplementary Figure 1** Sensitivity analysis of the association between refractive error and DR、VTDR.


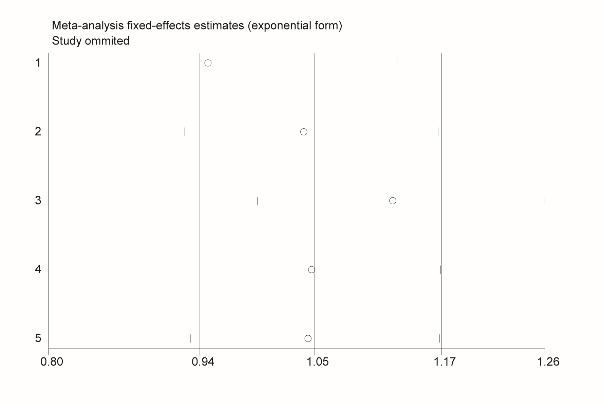

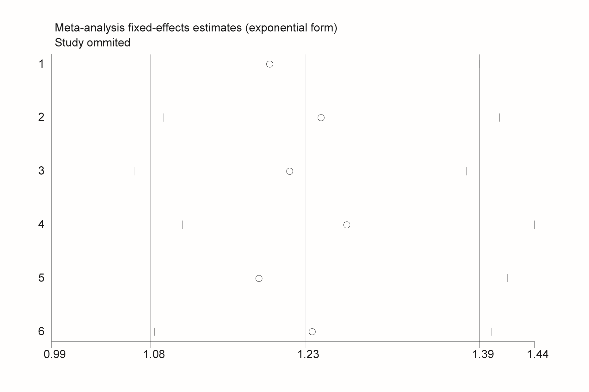


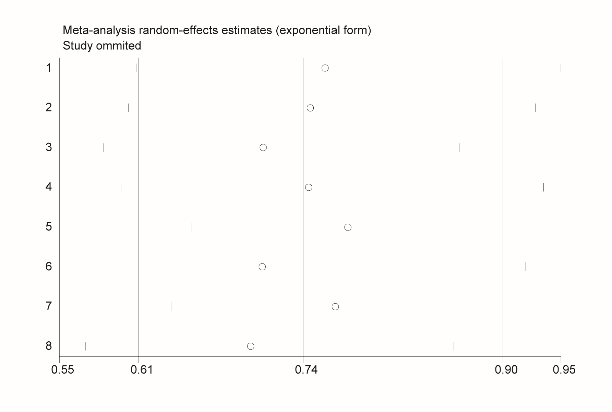

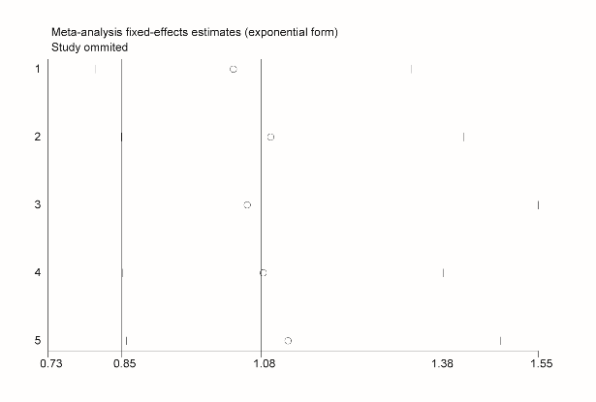


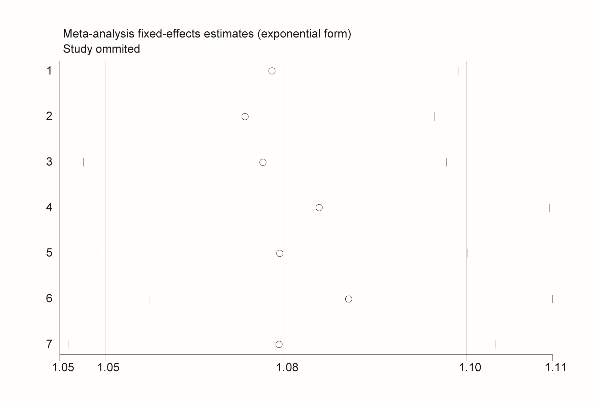

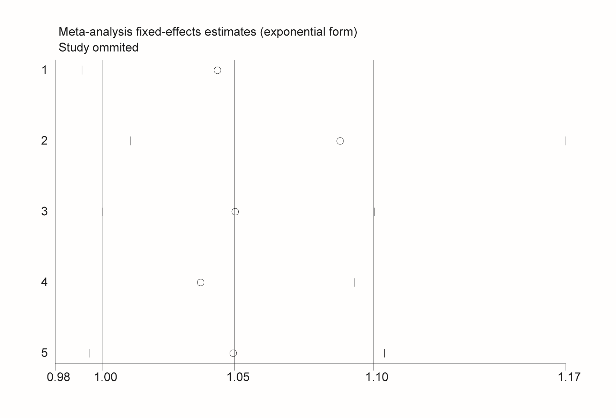


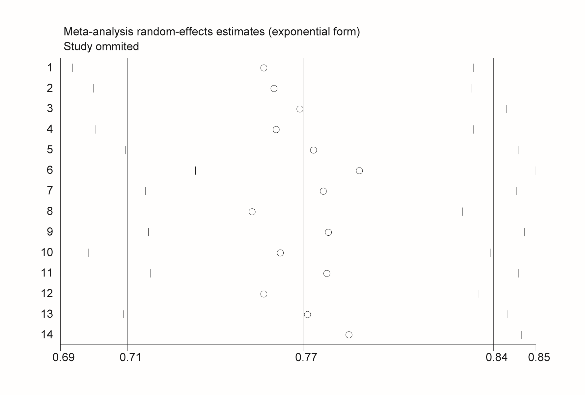

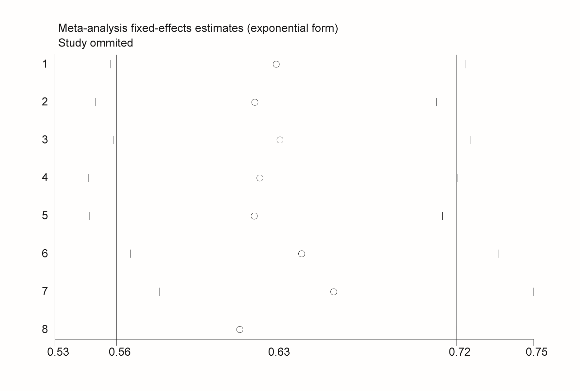


**Supplementary Figure 2.** Forest plot of the subgroup analysis on the association between myopia and DR risk.


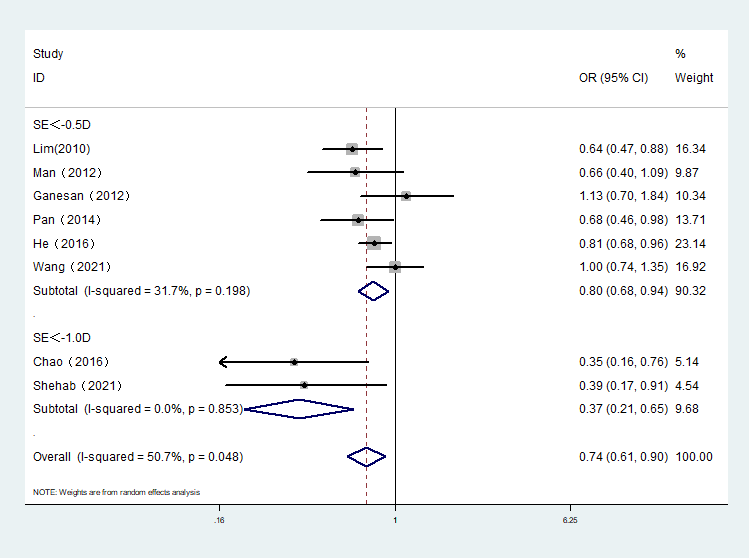


**Supplementary Figure 3.** Forest plot of the subgroup analysis on the association between AL (per mm increase) and DR risk.


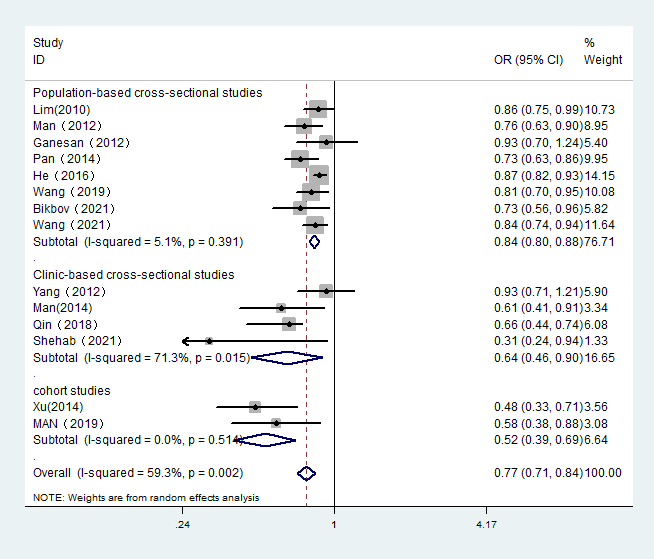


**Supplementary Figure 4.** The results of the trim and fill method in the model of AL and DR risk


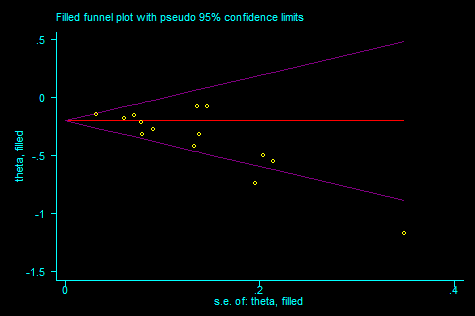


**Supplementary Table 1**  Search Strategy

| Subject headings ：  "Myopia, Hyperopia, Refractive Error, Axial Length, and Diabetic Retinopathy" |
| --- |
| Text words ：  "Myopia, Nearsightedness", "Hyperopia, Farsightedness", "Strabismus, Error, Refraction, Refractive Disorder, Disorder, Refraction", "Axial Lengths, Eye", "Eye Axial Length", "Length, Eye Axial", "Diabetic Retinopathies", "Proliferative DR", "Retinopathies, Diabetic" |
| Pubmed :  The database was searched on 30 June, 2023, n=296.  ((((("Refractive Errors"[Mesh]) OR (ametropia[Title/Abstract])) OR (("Myopia"[Mesh]) OR ((Myopias[Title/Abstract]) OR (Nearsightedness[Title/Abstract])))) OR (("Hyperopia"[Mesh]) OR ((Hypermetropia[Title/Abstract]) OR (Farsightedness[Title/Abstract])))) OR (("Axial Length, Eye"[Mesh]) OR ((axial length[Title/Abstract]) OR (Eye Axial Length*[Title/Abstract])))) AND (("Diabetic Retinopathy"[Mesh]) OR ((Diabetic Retinopathies[Title/Abstract]) OR (proliferative DR[Title/Abstract]))) |
| Embase：  The database was searched on 30 June, 2023, n=1316.  #1 'myopia'/exp myopias:ab,ti OR nearsightedness:ab,ti OR nearsightednesses:ab,ti  #2 'hypermetropia'/exp hypermetropia:ab,ti OR farsightedness:ab,ti  #3 'refraction error'/exp  ametropia:ab,ti OR 'error, refractive':ab,ti OR 'errors, refractive':ab,ti OR 'refractive error':ab,ti OR 'refractive disorders':ab,ti OR 'disorder, refractive':ab,ti  #4 'axial length'/exp 'axial lengths, eye':ab,ti OR 'eye axial length':ab,ti OR 'eye axial lengths':ab,ti OR 'length, eye axial':ab,ti OR 'lengths, eye axial':ab,ti  #5 #1 or #2 or #3 or #4  #6 'diabetic retinopathy'/exp 'diabetic retinopathies':ab,ti OR 'proliferative dr':ab,ti OR 'retinopathies, diabetic':ab,ti OR 'retinopathy, diabetic':ab,ti  #7 #5 and #6 |
| Cochrane Library：  The database was searched on 30 June, 2023, n=86.  #1 MeSH descriptor: [myopia] explode all trees  #2 (myopias or nearsightedness or nearsightednesses):ti,ab,kw  #3 MeSH descriptor: [Hyperopia] explode all trees  #4 (Hypermetropia or Farsightedness ):ti,ab,kw  #5 MeSH descriptor: [refraction error] explode all trees  #6 ( ametropia or error, refractive or errors, refractive or refractive error or refractive disorders or disorder, refractive ):ti,ab,kw  #7 MeSH descriptor: [axial length] explode all trees  #8 ( axial lengths, eye or eye axial length or eye axial lengths or length, eye axial or lengths, eye axial):ti,ab,kw  #9 #1 or #2 or #3 or #4 or #5 or #6 or #7 or #8  #10 MeSH descriptor: [diabetic retinopathy] explode all trees  #11 (diabetic retinopathies or proliferative dr or retinopathies, diabetic or retinopathy, diabetic ):ti,ab,kw  #12 #10 or #11  #13 #9 and #12 |
| Web of Science：  The database was searched on 30 June, 2023, n=899.  #1 Myopia (Topic) or Myopias (Topic) or Nearsightedness (Topic)  #2 Hyperopia (Topic) or Hypermetropia (Topic) or Farsightedness (Topic)  #3 Refractive Error (Topic) or ametropia (Topic) or Error, Refractive (Topic) and Disorder, Refractive (Topic)  #4 axial length (Topic) or Axial Lengths, Eye (Topic) or Eye Axial Length (Topic)  #5 #1 or #2 or #3 or #4  #6 Diabetic retinopathy (Topic) or Diabetic Retinopathies (Topic) or proliferative DR (Topic) and Retinopathies, Diabetic (Topic)  #7 #5 and #6 |
| CNKI：  The database was searched on 30 June, 2023, n=96.  (SU %= '近视' OR SU %= '远视' OR SU %= '屈光不正' OR SU %= '眼轴长度') AND SU %= '糖尿病性视网膜病变'  Translated to English as follows:  (SU %= 'Myopia' OR SU %= 'Hyperopia' OR SU %= 'Refractive Error' OR SU %= 'axial length') AND SU %= 'Diabetic retinopathy' |
| CBM：  The database was searched on 30 June, 2023, n=86.  ("糖尿病性视网膜病变"[常用字段:智能]) AND ("近视"[全部字段:智能] OR "远视"[全部字段:智能] OR "屈光不正"[全部字段:智能] OR "眼轴长度"[全部字段:智能])  Translated to English as follows:  ("Diabetic retinopathy"[Common Fields: Smart]) AND ("'Myopia"[Common Fields: Smart] OR "Hyperopia"[Common Fields: Smart] OR "Refractive Error"[Common Fields: Smart] OR "axial length"[Common Fields: Smart]) |
| Wan Fang Data：  The database was searched on 30 June, 2023, n=391.  ( (((主题=近视) OR 主题=远视) OR 主题=屈光不正) OR 主题=眼轴长度) AND 主题=糖尿病性视网膜病变  Translated to English as follows:  ( (((Theme=Myopia) OR Theme=Hyperopia) OR Theme=Refractive Error) OR Theme=axial length) AND Theme=Diabetic retinopathy |
| VIP databases：  The database was searched on 30 June, 2023, n=28.  M=(近视 OR 远视 OR 屈光不正 OR 眼轴长度) AND (M=糖尿病性视网膜病变)  Translated to English as follows:  M=(Myopia OR Hyperopia OR Hyperopia OR axial length) AND (M=Diabetic retinopathy) |
